# Effects of Micro-Arc Oxidation Discharge Parameters on Formation and Biomedical Properties of Hydroxyapatite-Containing Flower-like Structure Coatings

**DOI:** 10.3390/ma16010057

**Published:** 2022-12-21

**Authors:** Kuan-Ting Chen, Jun-Wei Huang, Wei-Ting Lin, Tsung-Yuan Kuo, Chi-Sheng Chien, Ching-Ping Chang, Yung-Ding Lin

**Affiliations:** 1 Department of Orthopaedics, Chi Mei Medical Center, No. 901, Zhonghua Rd., Yongkang District, Tainan 710, Taiwan; 2 Department of Mechanical Engineering, Southern Taiwan University of Science and Technology, 1 Nan-Tai St., Tainan 710, Taiwan; 3 Department of Medical Research, Chi Mei Medical Center, No. 901, Zhonghua Rd., Yongkang District, Tainan City 710, Taiwan; 4 School of Intelligent Engineering, Shaoguan University, Shaoguan 512005, China

**Keywords:** micro-arc oxidation, hydroxyapatite, flower-like structure, bioactivity, biocompatibility

## Abstract

The micro-arc oxidation (MAO) process was used to prepare hydroxyapatite-containing flower-like structure coatings on commercially pure titanium substrates with various values of the applied voltage (330, 390, 450 V), applied current (0.4, 0.5, 0.6 A), and duration time (1, 3, 5 min). It was found that the surface morphology of the coatings was determined primarily by the applied voltage. A voltage of 330 V yielded a flower-like/plate-like structure, while voltages of 390 V and 450 V produced a flower-like structure and a porous morphology, respectively. The applied current and duration time mainly affected the coating formation speed and petal size of the flower-like structures, respectively. The coatings prepared using voltages of 330 V and 390 V (0.6 A, 5 min) both contained Ti, TiO_2_-A (anatase), TiO_2_-R (rutile), DCPD (CaHPO_4_·2H_2_O, calcium hydrogen phosphate), and hydroxyapatite (HA). However, the latter coating contained less DCPD and had a higher HA/DCPD ratio and a Ca/P ratio closer to the ideal value of HA. The coating prepared with a voltage of 450 V consisted mainly of Ti, TiO_2_-A, TiO_2_-R, and CaTiO_3_. For the coatings prepared with a voltage of 390 V, the flower-like structures consisted mainly of HA-containing compounds. DCPD plate-like structures were observed either between the HA-containing flower-like structures (330 V samples) or within the flower-like structures themselves (390 V samples). The coating surfaces with flower-like/plate-like or flower-like structures had a greater roughness, which increased their hydrophilicity and resulted in superior bioactivity (SBF immersion) and biocompatibility (MG-63 cell culture). The optimal biomedical performance was found in the 390 V coating due to its flower-like structure and high HA/DCPD ratio.

## 1. Introduction

Titanium (Ti) and its alloys are widely used as biomedical implants due to their excellent mechanical properties, superior biocompatibility, and high corrosion resistance [1]. However, Ti and its native oxide thin film TiO_2_ are bio-inert, and thus, chemical bonds are not easily formed between the implants and the surrounding biological tissue. As a result, the implants have poor osteoconductivity and are often simply encased in fibrous tissue, thus prolonging the healing time [2]. Consequently, the long-term fixation of metallic implants in bone tissue remains an ongoing concern in the biomedical domain.

Hydroxyapatite (HA, Ca_10_(PO_4_)_6_(OH)_2_) is the main inorganic component of hard dental and bone tissues and has crystallographic and chemical properties similar to those of carbonated apatite [3]. Thus, HA coatings have been proposed as an effective surface modification technique for improving the osteoconductivity of metallic implants [2]. One of the most common methods for preparing HA coatings on metallic implants is the atmospheric plasma spraying process [4,5]. However, the spraying process is performed at extremely high temperatures (typically over 10,000 °C), and hence, the HA coating thermally decomposes into tricalcium phosphate (TCP in α/β form), tetracalcium phosphate (TTCP), calcium oxide (CaO), and amorphous phases [6,7]. TTCP and CaO phases have poor bioactivity and dissolve faster than other calcium phosphate phases when exposed to human body fluid [2]. Thus, the coating layer gradually flakes away from the implant surface, resulting in the release of deleterious metallic ions into the body and the eventual failure of the implant [8,9].

To overcome this problem, the literature contains many different proposals for depositing HA coatings on metallic implants, including sol gel synthesis [10,11], pulsed laser deposition [12,13], electrochemical reaction [14,15], and electrophoretic deposition [16,17]. Micro-arc oxidation (MAO) also provides an efficient approach for depositing porous biomedical coatings [18,19,20,21,22]. During the MAO process, a high-voltage micro-arc discharge event instantly raises the sample surface temperature to 2000–3000 °C, causing the surface to melt and regenerate into a thin layer of porous bioactive and biocompatible ceramic material with high hardness and suitable corrosion resistance [18,19,20,21,22]. Furthermore, MAO provides the opportunity to control the coating compounds (and hence the coating properties) by adjusting the electrolyte composition [23]. Notably, the MAO process is carried out in an electrolytic bath, making it ideal for the surface treatment of implants with complex geometries. Moreover, compared with plasma spraying techniques, MAO is simpler, requires a lower temperature, is more easily controlled, and is less expensive. As a result, it has many practical advantages as a coating technique. However, even though the crater-like structure formed by the MAO process yields a porous morphology, the surface roughness of the coating still needs to be improved to enhance the biomedical properties of the implant.

The flower-like structures that often form during the MAO process are crystals grown through oriented attachment, which preferentially nucleate at favorable locations. As the MAO time increases, these crystals spontaneously aggregate into flower-like agglomerates [24]. Compared with the volcano-like (crater-like) morphology of traditional MAO coatings, the flower-like morphology has a significantly greater surface roughness [25] and generates micro- or nano-structures which improve the biomedical properties of the implant surface [26]. Kossenko et al. [27] successfully prepared coatings with a flower-like structure on a Ti-6Al-4V substrate by combining MAO with hydrothermal treatment. However, the process was complicated and time-consuming. Consequently, Liu et al. [24,28,29] used a one-step MAO process to synthesize flower-like structure coatings on Ti substrates directly. However, the correlation between the flower-like structure formation and the discharge parameters was not systematically explored.

Accordingly, in this study, the MAO process was used to prepare HA-containing flower-like structure coatings on commercially pure titanium (Cp-Ti) substrates using various values of the main discharge parameters (namely the applied voltage, applied current, and duration time) and a mixed electrolyte solution consisting of calcium acetate ((CH_3_COO)_2_Ca·H_2_O)) and sodium dihydrogen phosphate (NaH_2_PO_4_·2H_2_O). The effects of the discharge parameters on the morphology, surface roughness, wettability, and biomedical properties of the various coatings were then systematically examined.

## 2. Materials and Methods

### 2.1. Preparation of Specimens

Commercially pure titanium disks (Grade 2, S-Tech Corp, Tainan, Taiwan) with dimensions of 14 mm (diameter) × 3 mm (thickness) were ground sequentially with 800- and 1000-grit SiC sandpaper, cleaned in acetone and distilled (DI) water, and then air-dried.

### 2.2. Preparation of Coatings by MAO Process

The MAO process was conducted in a mixed electrolyte solution consisting of 0.2 mol/L (CH_3_COO)_2_Ca·H_2_O and 0.1 mol/L NaH_2_PO_4_·2H_2_O. The Cp-Ti substrates were anodized with a DC power supply, and a stainless-steel electrode was placed at a distance of approximately 10 mm from the substrates to serve as a cathode. The MAO coating process was performed using three different values of the applied voltage (330, 390, 450 V), applied current (0.4, 0.5, 0.6 A), and duration time (1, 3, 5 min). For all of the coating processes, the electrolyte temperature was maintained at approximately 25 °C by a cooling system.

### 2.3. Measurement of Surface Roughness and Wettability (Contact Angle)

The average surface roughness (R_a_) of each coating surface was measured using a 3D laser scanning confocal microscope (3D-LSCM, VK-X200, KEYENCE Co., Osaka, Japan) based on a minimum of three separate measurements obtained at different positions on the coating surface. The wettability of the coatings was determined by the static sessile drop method using DI water as the working fluid. Contact angle (CA) measurement is often used to estimate the wettability of solid surfaces in contact with a liquid. The surface static CAs of the coating surfaces were measured using a CA goniometer (Theta Lite, Biolin Scientific, Sweden) with a 4 μL droplet volume and a stabilization time of at least 5 s. For each coating, the CA was measured three times in order to determine the mean CA value and corresponding standard deviation. 

### 2.4. In Vitro Bone-like Apatite Inducement Tests

The specimens were immersed in a standard simulated body fluid (SBF) solution [30] for 1, 3, and 7 days to assess the bone-like apatite inducement performance of the coatings. The SBF solution was maintained at a temperature of 37 ± 0.1 °C and was refreshed every other day. Table 1 compares the inorganic ion concentrations of the SBF solution with those of human blood plasma. Prior to the immersion process, the specimens were rinsed in DI water and disinfected with 75% alcohol.

### 2.5. Initial In Vitro Cell Culture Tests

The in vitro biocompatibility of the coatings was initially evaluated by inspecting the attachment and spreading of human MG-63 cells (Cat. No:CRL-1427, ATCC, Manassas, VA, USA) on the specimen surface. Briefly, cell suspensions were formed by digesting MG-63 cells in trypsin. The suspensions were seeded with a density of 5 × 103 cells/cm^2^ on MAO specimens placed in 12-well plates. The plates were maintained in Dulbecco’s Modified Eagle Medium (DMEM) supplemented with 10% fetal bovine serum (FBS) at 37 °C in a humidified incubator with 5% CO_2_ for 3 and 24 h. PBS buffer solution containing 2.5 vol % glutaraldehyde was utilized to fix the viable cells. Dehydration of the viable cells was carried out using a graded ethanol series (30%, 50%, 70%, 95%, and 100%). The cells were then critical-point-dried in hexamethyldisilazane (HMDS) for 10 min. 

### 2.6. Microstructure and Phase Composition

Scanning electron microscopy (SEM, JEOL JSM-6390LV, JEOL Ltd., Tokyo, Japan) was used to observe the microstructures of the various samples, and energy dispersive X-ray spectroscopy (EDS) was applied to evaluate the elemental compositions. Lastly, the phases’ compositions were identified by X-ray diffraction (XRD) with Cu Kα radiation (Rigaku D/Max III.V, Rigaku Ltd., Tokyo, Japan), a 2θ scanning range of 10°~60°, and a scanning rate of 3° min^−1^. 

## 3. Results and Discussion

### 3.1. Surface Morphology and Roughness of MAO Coatings

**Figure 1:**
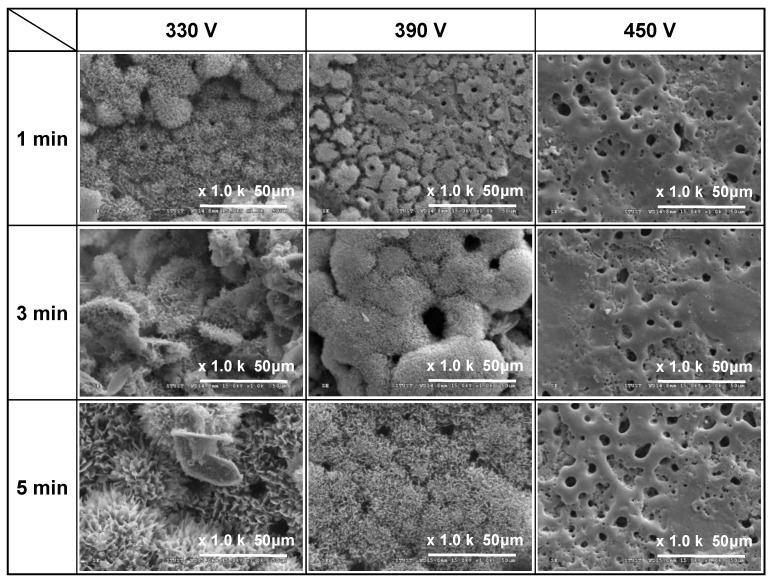
Surface morphologies of MAO coatings prepared with different applied voltages and duration times and constant applied current of 0.4 A.

**Figure 2:**
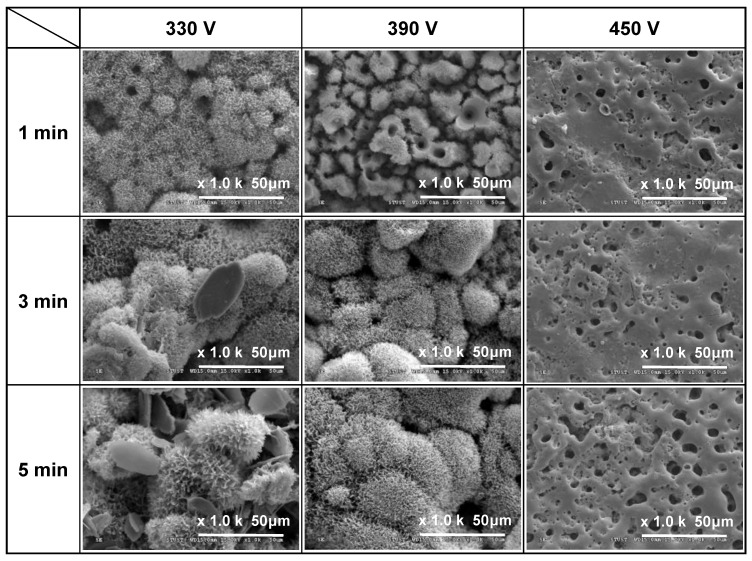
Surface morphologies of MAO coatings prepared with different applied voltages and duration times and constant applied current of 0.5 A.

**Figure 3:**
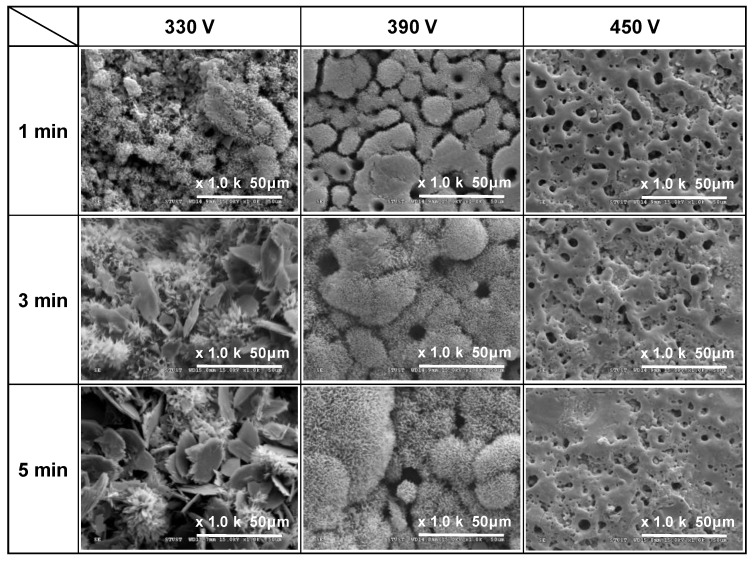
Surface morphologies of MAO coatings prepared with different applied voltages and duration times and constant applied current of 0.6 A.

Figure 1, Figure 2 and Figure 3 show the SEM morphologies of the coating surfaces prepared using different applied voltages (330–450 V) and duration times (1–5 min) with fixed currents of 0.4, 0.5, and 0.6 A, respectively. It is seen that all of the 330 V and 390 V samples (with the exception of the 390 V, 1 min sample) have flower-like structures and micropores on the coating surface. Moreover, the size of the petals and pores increases with an increasing duration time. However, the 450 V samples have a typical MAO porous morphology without flower-like structures. In particular, the surfaces have a crater-like volcanic appearance due to the electrical breakdown and surface reconstruction of the weak part of the Ti surface [29].

For the samples prepared with a voltage of 330 V for 3 min and 5 min, additional plate-like structures are formed, which gradually increase in size and number with an increasing current and MAO duration time. For the coating prepared with the longest duration time of 5 min and the lowest applied current of 0.4 A, the ratio of the flower-like structures to the plate-like structures is around 9:1. However, for the maximum applied current of 0.6 A, the number of plate-like structures exceeds that of the number of flower-like structures by a ratio of around 4:6. Generally, the results presented in Figure 1, Figure 2 and Figure 3 show that under the discharge parameters considered in the present study, a large number of micropores with diameters of approximately 1–25 μm are formed on the coating surface. Notably, many studies have shown that pore sizes smaller than 15–50 μm are beneficial for promoting fibrovascular ingrowth [31,32] and nutrient circulation [33] on biomedical implant materials.

**Figure 4:**
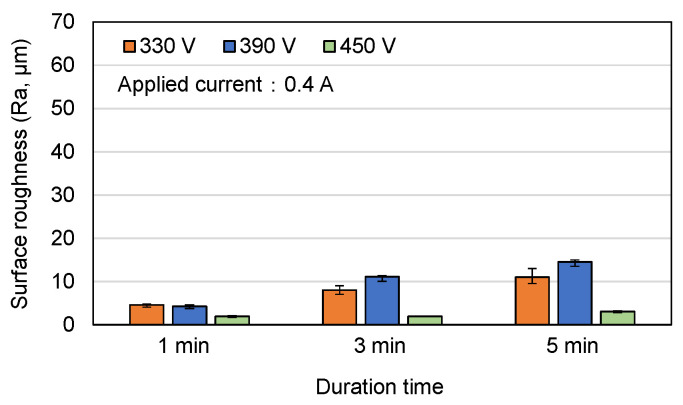
Surface roughness of MAO coatings prepared with different applied voltages and duration times and constant applied current of 0.4 A.

**Figure 5:**
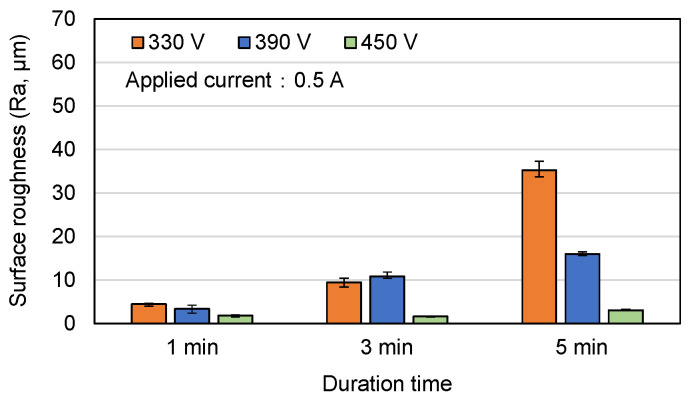
Surface roughness of MAO coatings prepared with different applied voltages and duration times and constant applied current of 0.5 A.

**Figure 6:**
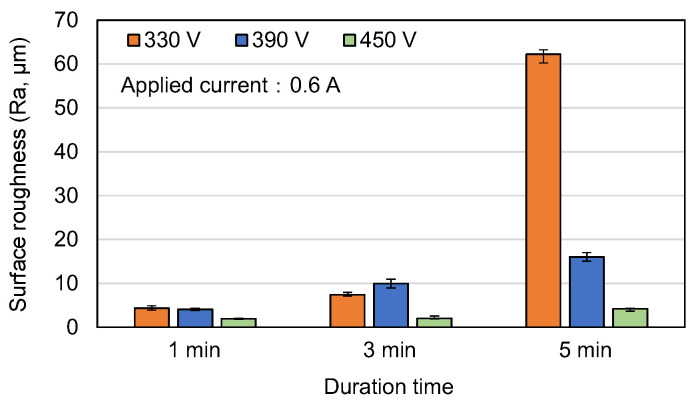
Surface roughness of MAO coatings prepared with different applied voltages and duration times and constant applied current of 0.6 A.

Figure 4, Figure 5 and Figure 6 show the surface roughness (R_a_) values of the MAO samples in Figure 1, Figure 2 and Figure 3. In general, the results show that the surface roughness of the coatings with flower-like or plate-like structures (330 V, 390 V) increases with an increasing current and duration time. For the coatings prepared using the longest duration time of 5 min, the surface roughness increases with a reducing voltage. This tendency is particularly apparent under higher values of the applied current. For example, the R_a_ values of the 330 V and 390 V samples are 35.0 μm and 15.0 μm, respectively, for an applied current of 0.5 A, but increase to 62.2 μm and 16.0 μm, respectively, for an applied current of 0.6 A. This result is reasonable since the 330 V (3 min, 5 min) sample consists mainly of plate-like structures, which are larger in size than the flower-like structures, and the size of these structures increases with an increasing current, leading to a greater surface roughness. For the 450 V samples (with a typical MAO porous morphology), the surface roughness is significantly lower than that of the 330 V and 390 V samples for all values of the applied current and MAO duration. From inspection, the surface roughness lies in the range of Ra = 1.9~3.0 μm and varies insignificantly with changes in the MAO processing conditions.

In general, surface roughness can be classified into three levels according to the size of its surface features, namely nanoscale (1–100 nm), microscale (1–10 μm), and macroscale (10 μm-mm) [34]. For the 330 V and 390 V samples, the surface roughness falls in the macroscale range, while for the 450 V sample, it lies in the microscale range. Compared to smooth surfaces, coatings with a macro-sized topography result in significantly improved early implant fixation and provide an improved long-term mechanical stability of prostheses since the higher surface roughness creates a better interlocking effect between the implant surface and the bone ongrowth [34,35].

The results presented in Figure 1, Figure 2 and Figure 3 show that the formation of flower-like structures in the MAO coatings is determined primarily by the discharge voltage. By contrast, the current and duration time play more minor roles. The effects of the individual MAO discharge parameters on the formation of flower-like structures are briefly described in the following.

Effects of applied voltage (330~450 V): Taking the samples prepared with an applied current of 0.5 A (Figure 2) for illustration purposes, it is seen that an applied voltage of 330 V for 1 min results in the formation of a flower-like structure. However, for a higher voltage of 390 V, only island-like structures are formed after 1 min, and the duration time must be increased to 3 min to form flower-like structures. In other words, the flower-like structures are more easily formed at lower applied voltages. Previous studies have shown that for a constant sample-to-cathode distance, the micro-arc energy (i.e., the temperature) reduces as the voltage reduces [36]. Consequently, the lower applied voltage of 330 V in the present study provides a more stable growth environment for relatively fine flower-like structures. However, as the applied voltage increases, the temperature also increases and hence the coating surface cannot form a flower-like structure. Thus, the coating morphology has a relatively rough island-like characteristic (390 V, 1 min) or a typical MAO structure (450 V).

Effects of applied current (0.4~0.6 A): Taking the samples prepared with an applied voltage of 390 V (Figure 1, Figure 2 and Figure 3) for illustration purposes, it can be seen that, regardless of the MAO processing time, the coating structure coarsens slightly with an increasing current, and an obvious island-like structure is formed after 1 min. In other words, the application time is 1 min. This indicates that the applied current has a significant effect on the growth rate of the coating structure. This finding is reasonable since a higher applied current induces a greater current density, which reduces the time required to reach the oxide breakdown voltage. As a result, the micro-arc discharge event occurs earlier, and the growth rate of the coating increases accordingly [37,38].

Effects of duration time (1~5 min): Taking the coatings prepared with an applied voltage of 330 V and current of 0.6 A (Figure 3) for example purposes, it is found that the average size of the flower-like structure of the coatings increases from 2.68 μm to 6.98 μm as the MAO duration time increases from 1 to 5 min. This finding is again reasonable since a longer MAO duration increases the reaction time of the coating and therefore provides more energy for the continuous growth of the flower-like structures [39,40,41]. Previous studies [42] have also shown that in conventional MAO porous coatings, the coating thickness increases significantly with an increasing processing time. In general, the SEM images in Figure 1, Figure 2 and Figure 3 indicate that for the present coatings, an increasing MAO processing time not only increases the ratio of the flower-like to plate-like structures, but also increases the petal size of the flower-like structures.

### 3.2. EDS and XRD Analysis Results for MAO Coating Surfaces

The results presented in the previous section have shown that among all of the present MAO coatings, the morphologies of the coatings prepared with a current of 0.6 A and duration of 5 min are the most sensitive to the applied voltage. Accordingly, the 0.6 A/5 min series of coatings were selected for further detailed surface chemical and phase composition analysis.

**Figure 7:**
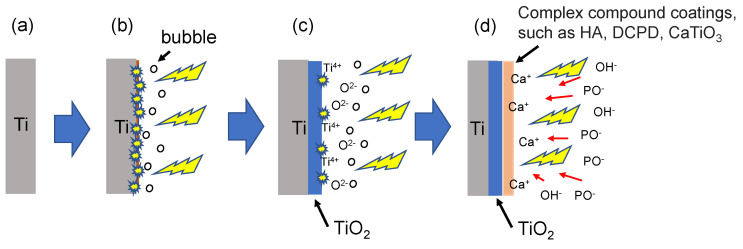
Schematic illustration of coating development during micro-arc oxidation discharge process on titanium substrate (**a**) Stage 1, (**b**) Stage 2, (**c**) Stage 3 and (**d**) Stage 4.

Figure 7 presents a schematic illustration of the MAO coating development during the MAO discharge process on a Ti substrate. The reaction characteristics (i.e., development process) of the MAO coating can be roughly divided into four stages. Stage 1: A passivation oxide layer is formed on the surface of the original (Cancel) Ti substrate (**a**). Stage 2: When an appropriate electrical potential is applied to the substrate, the passivation layer begins to grow, forming a channel as the voltage increases. When the voltage reaches a certain critical value, a micro-arc begins to be generated in the channel. In addition, the water in the electrolyte is electrolyzed and generates bubbles (**b**). Stage 3: As the voltage continues to increase, the oxidation rate of the coating gradually increases, and the weaker oxide layer is broken down by the intense micro-arc discharge. A new oxide layer grows in its place, and the dissociated Ti^4+^ cations, subject to high temperature and pressure conditions, react with the electrolytic oxygen anions to form porous TiO2 film, which gradually spreads over the entire surface (**c**). Stage 4: Under continuous discharge, all of the ions in the electrolyte, including OH-, PO_4_^3−^, HPO_4_^2−^, and Ca^2+^, diffuse and migrate to the anode under the effects of electrophoresis, where they may subsequently form complex compounds (**d**). (Note that a more detailed description of the discharge process is available in references [16,24,39].)

**Figure 8:**
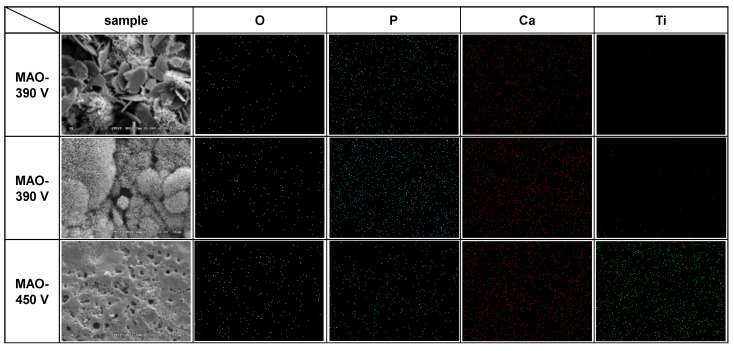
EDS elemental mappings and corresponding SEM images (applied current: 0.6 A, duration time: 5 min).

**Figure 9:**
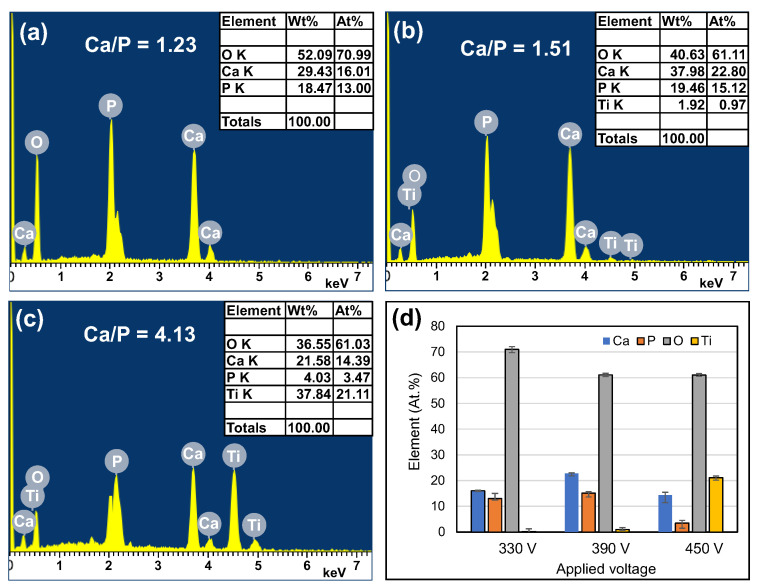
EDS spectra at accelerating voltage of 15 kV for (**a**) 330 V, (**b**) 390 V, and (**c**) 450 V coatings. (**d**) Average chemical compositions of (**a**–**c**) (applied current: 0.6 A, duration time: 5 min).

**Figure 10:**
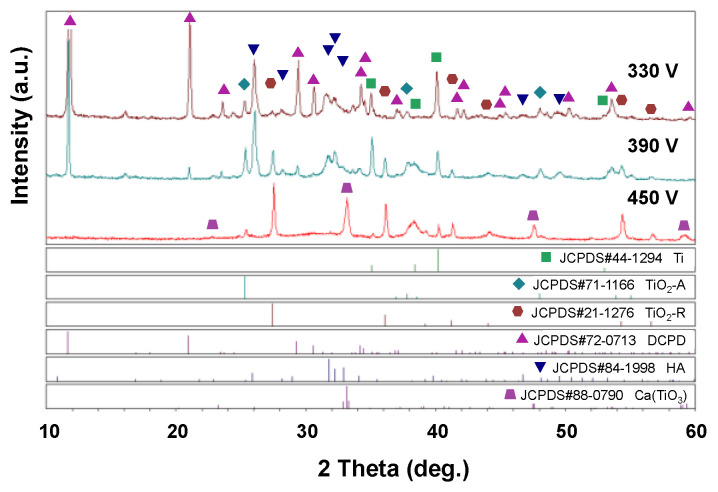
XRD patterns of MAO coatings prepared with different applied voltages (applied current: 0.6 A, duration time: 5 min).

Figure 8 shows the EDS elemental mapping results and SEM surface images for the three samples processed with applied voltages of 330 V, 390 V, and 450 V at 0.6 A/5 min. Figure 9 and Figure 10 show the EDS analysis results and XRD patterns, respectively, of the sample surfaces shown in Figure 8. The EDS mapping images in Figure 8 show that there is almost no Ti in the 330 V sample and only a small quantity of Ti in the 390 V sample. The 450 V sample contains a large quantity of evenly distributed Ti, but has a significantly reduced quantity of P. The overall distributions of the other Ca, P, and O elements are uniform, and no aggregation phenomenon is observed. For the 330 V sample (Figure 9a), the EDS analysis results show that the coating consists predominantly of Ca, P, and O, which are the main components of bone-like apatite. No Ti element (substrate) is observed. The corresponding XRD pattern (Figure 10) shows peaks corresponding to Ti, TiO_2_-A (anatase), TiO_2_-R (rutile), DCPD (brushite, CaHPO_4_·2H_2_O, dicalcium phosphate dihydrate), and HA. For the 390 V sample (Figure 9b), the main coating elements are the same as those of the 330 V sample, namely Ca, P, and O. However, a trace amount of Ti is also detected. As shown in Figure 10, the phase composition of the 390 V coating is similar to that of the 330 V sample. However, the 390 V sample shows higher diffraction peaks of TiO_2_-R and a lower diffraction peak of DCPD. In addition, in the angle range of the main diffraction peak of HA (2θ = 30.5–31.5°), the peak of the 390 V sample is slightly higher and sharper than that of the 330 V sample, indicating that its HA content and crystallinity are slightly higher than those of the 330 V sample, and are thus expected to enhance the bioactivity [42]. Furthermore, the 390 V sample has a lower content of DCPD (which is less bioactive than HA), and thus has a higher HA/DCPD ratio, making its Ca/P ratio closer to the ideal value of HA (1.67). All of these factors suggest that the 390 V sample has a higher bioactivity than the 330 V sample. Many studies on MAO have reported that flower-like HA or HA/TiO_2_ is formed under appropriate discharge conditions [24,28]. Comparing the morphologies, EDS compositions, and XRD spectra of the present coatings, it is inferred that the flower-like structures in the 330 V and 390 V samples are mainly HA-containing compounds, and the coatings have Ti substrate oxide in the inner layer [28].

DCPD is a metastable compound and is often observed as a primary crystalline product when calcium phosphate is precipitated under low temperature and pH conditions [43,44,45]. Furthermore, DCPD crystals are easily formed by direct synthesis in phosphate solutions and generally have the form of plate-like structures [46]. Han and Liu [28,47] prepared CaP coatings using the MAO process and found that they reacted to form DCPD-containing structures under appropriate discharge parameters. Liu et al. [28] showed that as the applied voltage increased, the micro-arc discharge intensified and increased the temperature and pressure in the micro-arc area accordingly. This in turn increased the driving force of diffusion and electrophoresis, which accelerated the migration of the OH^−^, PO4^3−^, HPO_4_^2−^, and Ca^2+^ ions in the electrolyte and resulted in the formation of a HA coating through the reaction of DCPD with the OH^−^ ions. Previous studies [43,48] have also shown that biological apatite can be formed by the dissolution (hydrolysis) of disordered DCPD to obtain stable HA through a rapid solid–solid phase transition. This HA product has been identified as calcium-deficient HA [49]. DCPD is thus considered as one of the main precursors of the apatite phase that constitutes the mineral composition of bone [47].

The plate-like structures observed in the coatings prepared using an applied voltage of 330 V and durations of 3 min and 5 min were analyzed by EDS and XRD and compared with the structures reported in previous studies [28,47]. The results showed that the plate-like structures were DCPD. Although no plate-like structures were observed on the surface of the 390 V sample, DCPD diffraction peaks were nevertheless apparent in the XRD pattern, albeit with an intensity far lower than that of the peaks in the pattern of the 330 V sample. Moreover, the intensity of the HA diffraction peaks was increased. The higher HA peak intensity is thought to arise since, when the discharge energy in the MAO process increases, the hydrolysis of the HA precursor (DCPD) in the coating also increases [45,48], particularly that of the DCPD on the coating surface. Thus, DCPD does not readily grow on the surface of the coating, and the higher discharge energy converts the unhydrolyzed DCPD into HA [28]. Consequently, although the coating surface does not show a plate-like structure, a small quantity of DCPD resides within the coating, and hence weak diffraction peaks are observed in the XRD pattern. Overall, the results indicate that, depending on the discharge voltage, the DCPD lies either between the HA-containing flower-like structures (330 V sample) or within the flower-like structures themselves (390 V sample).

As shown in Figure 9c, the main chemical components of the 450 V coatings include Ca, P, O, and Ti (with the Ti content to 21.11 at %). Moreover, the phase compositions include Ti, TiO_2_-A, TiO_2_-R, and calcium titanate (CaTiO_3_), as shown in Figure 10. The main difference between the 450 V sample and the 330 V and 390 V samples is thus the absence of HA and DCPD phases, and the inclusion of CaTiO_3_ phase. In the MAO process, a higher applied voltage increases the reaction intensity, and thus favors the formation of TiO_2_-R (high temperature stable phase) over that of TiO_2_-A (low temperature stable phase). In addition, in the center of the spark zone, the temperature is sufficiently high (3000~10,000 K [20]) to melt the oxide [50], and this favors the incorporation of the Ca^2+^ and OH^−^ ions in the electrolyte into the TiO_2_ in the coating. As a result, the quantity of TiO_2_-R increases and promotes the formation of CaTiO_3_ [51].

In general, MAO coatings deposited on Ti substrates under certain conditions (such as those used in the present study) form both inner and outer layers. The inner layer is formed by the oxidation of the Ti substrate (such as TiO_2_-A and TiO_2_-R), while the outer layer is formed by the reaction of the inner layer with the MAO electrolyte (such as DCPD, HA, CaTiO_3_). (Note that the detailed formation mechanism of the composite coating is available in [28].) According to the formation mechanism of the coating, it is speculated that the Ti peak (2θ = 40°) observed in the present XRD patterns comes from the substrate. However, the thickness of the 330 V, 390 V, and 450 V coatings (around 9.7, 18.2, and 29.1 µm, respectively) increases with an increasing applied voltage, and hence the intensity of the Ti peaks decreases.

The Ca/P ratio (Figure 9a,b) of the MAO coatings increased from 1.23 in the 330 V sample to 1.51 in the 390 V sample. In general, the Ca/P ratios of DCPD and HA are stoichiometrically 1 and 1.67, respectively [52]. In other words, the Ca/P ratio increases with an increasing discharge voltage, and part of the calcium phosphate phase transforms from DCPD to HA. However, the transformation is incomplete, and hence the Ca/P ratio of the 390 V sample does not reach 1.67. For the 450 V sample, the P content is relatively low (Figure 9c) due to the absence of obvious calcium phosphate phase formation, and thus the Ca/P ratio has a high value of 4.13.

### 3.3. Wettability of MAO Coatings

**Figure 11:**
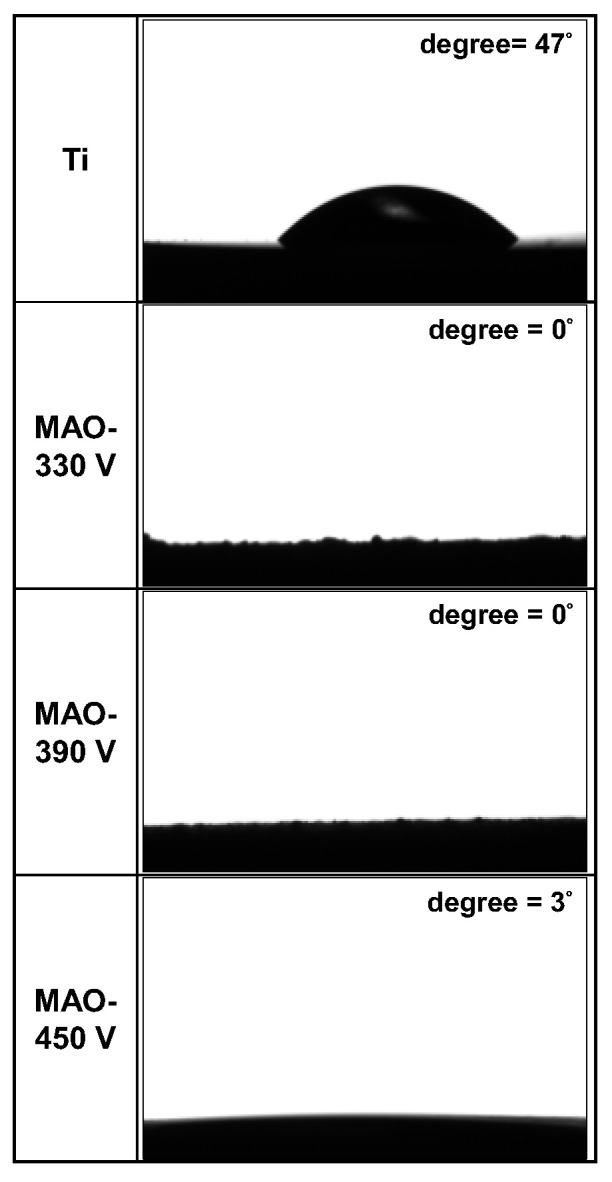
Photographs of deionized water droplets on Ti substrate and MAO coatings prepared with different applied voltages (applied current: 0.6 A, duration time: 5 min).

Figure 11 shows the CA values of the coatings prepared with different MAO processing parameters. A lower CA value indicates an improved hydrophilicity and thus a better wettability. The original ground Ti surface has a CA of around 47°. By contrast, the 330 V and 390 V samples, with flower-like/plate-like and flower-like surface structures, respectively, have CA values close to 0°, while the 450 V sample has a CA value of approximately 3°. In other words, the original Ti substrate has adequate hydrophilicity, while the MAO coatings (particularly those processed at 330 V and 390 V) have super-hydrophilicity.

The CA of liquid droplets on solid substrates depends mainly on the surface topography (especially the surface roughness) and the surface chemistry [53,54,55]. In terms of the surface topography, for hydrophilic surfaces, an increased surface roughness leads to a lower CA, indicating a more hydrophilic nature. In the present study, the 330 V and 390 V samples exhibit super-hydrophilicity due to the large surface area provided by their higher surface roughness (Figure 6). In terms of the surface chemistry, the -OH and PO_4_^3−^ groups of the HA contained in the 330 V and 390 V coatings are hydrophilic. They thus readily form hydrogen bonds with H_2_O and induce a spreading of the water droplet on the coating surface [29]. For the 450 V sample, the coating contains neither flower-like nor plate-like structures, and its roughness (Figure 6) is much lower than that of the 330 V and 390 V samples. Furthermore, Rao et al. [51] reported that CaTiO_3_ phase, one of the main components of the 450 V coating, has poor hydrophilicity. Thus, the hydrophilicity of the 450 V sample is slightly poorer than that of the 330 V and 390 V samples.

Several studies have shown that TiO_2_ phase may be either hydrophobic (CA > 90°) or hydrophilic (CA < 90°), depending on the type of surface treatment process employed and the parameters used [52,56,57,58]. Although there are many relevant factors affecting the wettability of TiO_2_-containing coatings, one of the most critical factors is the OH group content of the coating. In general, a higher OH group content is associated with an improved hydrophilicity. As reported previously [59,60], the formation of OH is ascribed to the dissociation of TiO_2_ to H_2_O. Chu et al. [61] also showed that higher discharge voltages favor the formation of OH groups on the surface of the coating during MAO processing. Therefore, in the present study, it can be inferred that since the TiO_2_ content of the MAO coatings promotes the formation of OH groups, it mitigates the poor hydrophilicity of the CaTiO_3_ phase and improves the hydrophilicity of the coating surface as a result. Thus, under the combined effects of the topography and surface chemistry, the 450 V coating has a much better hydrophilicity than the original Ti substrate. Notably, a higher hydrophilicity (i.e., a lower CA) is beneficial in inducing osteoblast growth and mineral deposition [58,61].

### 3.4. In Vitro Bioactivity of MAO-Formed Coatings

**Figure 12:**
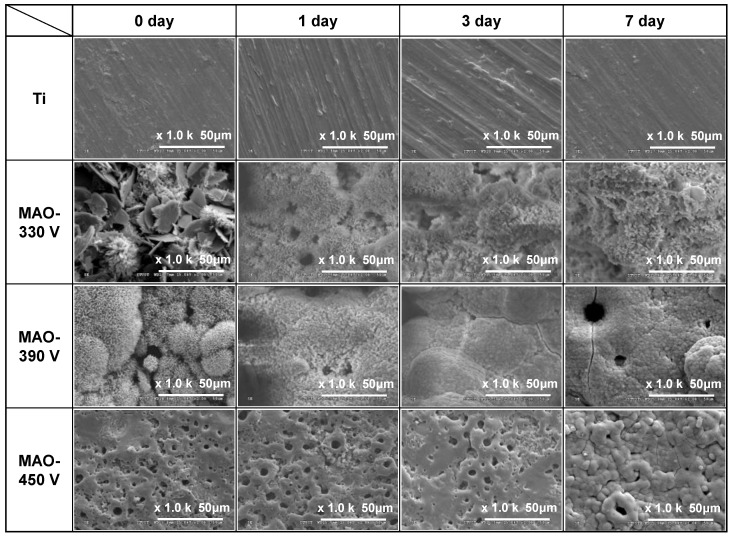
SEM micrographs of Ti substrate and MAO coatings prepared with different applied voltages after soaking in SBF for 1, 3, and 7 days (applied current: 0.6 A, duration time: 5 min).

**Figure 13:**
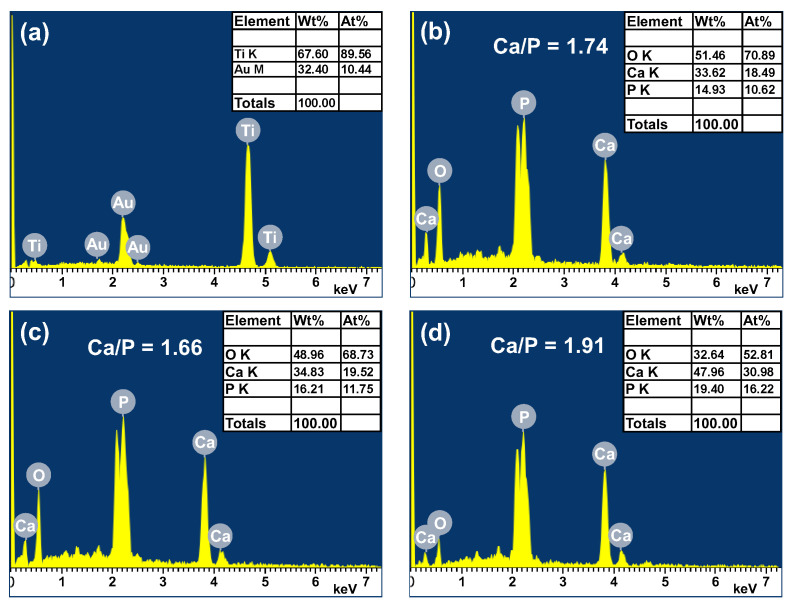
XRD analysis results for (**a**) Ti substrate, (**b**) 330 V, (**c**) 390 V, and (**d**) 450 V coatings after soaking in SBF for 7 days (applied current: 0.6 A, duration time: 5 min).

The in vitro bioactivity of the MAO coatings (0.6 A/5 min) was investigated by immersing the samples in an SBF solution with the same ionic composition as that of human plasma (Table 1) and observing the growth of apatite on the coating surface after 1, 3, and 7 days. After immersion for one day, only the 390 V coating showed particle (i.e., apatite) nucleation (Figure 12). After 3 days of soaking, the 330 V coating also showed evidence of particle nucleation and the 390 V coating was completely covered with particles. However, no particles were formed on the 450 V coating. After 7 days, the surfaces of all three coatings were almost completely covered with particles. However, no particles were formed on the surface of the original Ti substrate regardless of the soaking time. Figure 13 presents the EDS analysis results for the substrate and MAO coatings after soaking in SBF for 7 days. The results show that the coatings were mainly composed of Ca, P, and O, which are the main components of bone-like apatite [62]. The Ca/P ratio of the 330 V and 390 V samples increased from 1.23 to 1.74 and 1.51 to 1.66 after soaking, respectively (Figure 9). For both coatings, the Ca/P molar ratio is close to 1.67, which corresponds to the stoichiometric ratio of HA. In other words, when the coatings are immersed in SBF, HA is continuously deposited and aggregated on the coating surface. Moreover, as the Ca/P ratio approaches 1.67, a dense and thick HA coating is produced. The formation of apatite on the 450 V sample is slower than that on the other samples. In addition, its Ca/P ratio decreased from 4.13 before immersion to 1.91 after 7 days. Hence, the Ca/P ratio deviates slightly from the ideal value of 1.67. However, as shown in Figure 10, the coating was still covered with apatite after soaking for 7 days and thus still showed adequate bioactivity.

The plate-like DCPD in the 330 V coating is thermodynamically unstable in SBF [47] and is roughly dissolved after immersion in SBF for 1 day. This leads to the production of additional Ca and PO_4_^3−^ ions in the SBF, which promote the formation of apatite [63]. Consequently, apatite particles begin to nucleate on the coating surface after 3 days and subsequently grow and transform into HA [64]. Although the HA peak of the 390 V coating is not significantly higher than that of the 330 V coating, it contains less DCPD (with low bioactivity) and has a slightly higher crystallinity. Consequently, apatite is formed more readily on its surface following immersion in SBF. The 450 V coating contains no calcium phosphate phase. However, its main compound, CaTiO_3_, is hydrolyzed when immersed in SBF, which results in the generation of a large number of Ti-OH groups [51,65]. These groups trigger the absorption of PO_4_^3−^ by the Ca^2+^ ions in the SBF solution, which contributes to the electrostatic potential interaction between the apatite nuclei and the surface of the coating [66,67]. In addition, the TiO_3_^2−^ in CaTiO_3_ has the effect of attracting the Ca and P ions from the SBF to promote the nucleation of apatite [68]. Both mechanisms greatly promote the nucleation and growth of apatite, and hence the surface of the 450 V coating is also covered by a dense layer of apatite after immersion for 7 days. Previous studies on the MAO process have shown that coatings containing TiO_2_-A or TiO_2_-R have poor apatite growth ability in SBF, even following prolonged immersion times of 14 [65] or 28 days [69]. That is, TiO_2_-A or TiO_2_-R have no obvious benefit on bioactivity.

### 3.5. Initial In Vitro Biocompatibility of MAO-Formed Coatings

**Figure 14:**
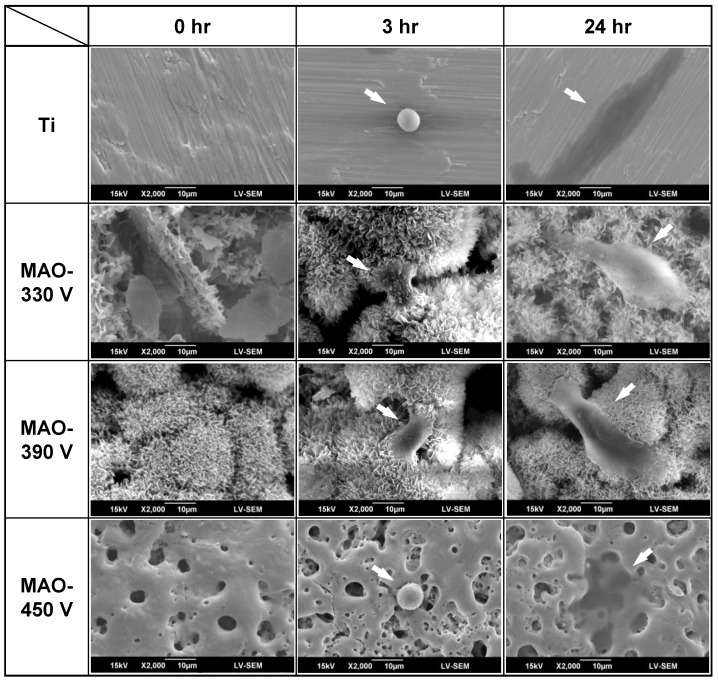
SEM micrographs of MG-63 cell morphology on Ti substrate and MAO coatings prepared with different applied voltages after incubation periods of 0–24 h (applied current: 0.6 A, duration time: 5 min).

In vitro biocompatibility tests were performed by seeding MG-63 osteoblast-like cells on the sample surfaces. The subsequent cell adhesion and growth behavior of the cells was then examined using SEM. Figure 14 shows the attachment and spreading of the cells on the surface of the bare Ti substrate and MAO coatings after 0, 3, and 24 h of incubation. In general, cell attachment and spreading can be classified into three main orders: (a) not spread, the cells have a spherical appearance; (b) partially spread, the cells begin to spread laterally on one or more sides; and (c) fully expanded, the plasma membrane extends to all sides, the spreading area enlarges, and the cells show a marked flattening effect [54]. After 3 h of incubation, the cells on the 330 V and 390 V coatings, with flower-like/plate-like and flower-like structures, respectively, are partially spread on the coating surface and show protruded filopodia. By contrast, for the bare Ti substrate and 450 V coating, the cells retain their original spherical appearance. After 24 h, the cells on the 330 V and 390 V samples have an expanded and flattened appearance, and those on the 390 V surface exhibit pronounced protruded filopodia. The cells on the 450 V sample and Ti surface also show marked cell expansion, but no filopodia. Overall, the results indicate that, in terms of their in vitro biocompatibility, the samples can be ranked as follows: 390 V > 330 V > 450 V > Ti substrate.

In general, cell growth behavior is strongly influenced by the surface properties, including the surface composition, roughness, hydrophilicity, texture, and morphology [29,53,70,71,72,73,74]. Among these properties, the surface roughness and composition are considered to be the most important parameters affecting cell viability [53,72]. For the present MAO coatings, the flower-like structures in the 330 V and 390 V coatings increase the surface roughness and hydrophilicity. This increases the surface area of the coatings and the number of potential attachment sites of the cells, and hence improves the biocompatibility [29].

It is generally held that a smaller CA value indicates a better hydrophilicity, which is beneficial to cell adhesion. However, the exact nature of the relationship between the CA value and the degree of cell adhesion remains unclear. Previous studies have yielded conflicting findings in this regard [75,76], most probably due to the fact that the cell adhesion performance is affected by multiple factors, including the surface morphology, the chemical properties (i.e., hydroxyl groups), the compounds present on the sample surface, and so on. Many studies have reported that CaTiO_3_ improves biocompatibility [77,78] and promotes osteoblast adhesion [79]. However, although CaTiO_3_ coatings have sufficient biocompatibility to be used as bioimplant coatings, Wu et al. [68] showed that CaTiO_3_ has weaker cell adhesion than HA and thus takes longer to achieve suitable adhesion between the implant and the bone. DCPD provides an appropriate microenvironment for the adhesion and growth of osteoblasts [80], but its response to different cell lines is significantly different [80,81]. Furthermore, the solubility of DCPD in cell culture medium is relatively higher than that of HA. Thus, in the early stage of cell culturing, the surface state is less stable and is hence less conducive to the adhesion of filopodia [47,82]. In addition, the apatite composition of HA is similar to that of bone tissue and thus offers suitable biocompatibility. As a consequence, HA-containing flower-like structure coatings not only exhibit a superior structure (with more ridges and corners), but also provide more adhesion sites for cells (provided by HA) [29]. Therefore, the 330 V and 390 V samples show a better cell adhesion and spreading performance than the 450 V samples. However, compared to the ground surface of the Ti substrate, all of the MAO coatings provide a rougher, calcium phosphate-containing, superhydrophilic surface, and therefore provide more adhesion sites for osteoblasts to facilitate cell adhesion and spreading.

During cell culturing, when the surface comes into contact with the biological tissue, water molecules first reach the surface. Therefore, the surface topography plays an important role in determining the cell adhesion behavior [83,84]. In addition, surface wettability at this stage also plays a major role in governing protein adsorption to the surface as well as cell adhesion. In particular, adequate surface wettability (low CA) not only improves the attachment between cells and host tissues after implantation [85], but also enhances the adsorption of proteins [86]. However, the subsequent proliferation of the attached cells depends on the toxicity (or otherwise) of the sample surface, which is largely determined by the surface chemistry [54]. Based on the findings of previous studies [29,47,53,54,70,71,72,73,74,75,76,77,78,79,80,81,82,83,84,85,86], the present results suggest that when the cell culture time is prolonged, HA-containing flower-like structure coatings not only instigate initial cell attachment, but also promote the subsequent proliferation and differentiation of the cells. The bioactivity and biocompatibility of the coatings are thus significantly better than those of traditional MAO samples and bare titanium substrates.

## 4. Conclusions

In the present study, we used the MAO technique to prepare HA-containing coatings on commercially pure titanium (Cp-Ti) substrates using various values of the discharge voltage, discharge current, and duration time, respectively, and a mixed electrolyte solution consisting of calcium acetate ((CH_3_COO)_2_Ca·H_2_O) and sodium dihydrogen phosphate (NaH_2_PO_4_·2H_2_O). The experimental results support the following major conclusions.

1. Among the various discharge parameters considered in this study, the applied voltage had the greatest effect on the surface structure and morphology of the coatings. In particular, the 330 V coating had a mixed flower-like/plate-like structure, while the 390 V and 450 V coatings had a flower-like structure and a typical MAO porous morphology, respectively. The applied current mainly determined the coating formation speed, while the duration time governed the petal size of the flower-like structure. Among all the samples, the 0.6 A/5 min samples showed the greatest variation in the coating surface morphology with changes in the discharge voltage.
2. The 330 V and 390 V coatings prepared with an applied current of 0.6 A and duration time of 5 min consisted mainly of Ti, TiO_2_-A, TiO_2_-R, DCPD, and HA. Of the two coatings, the 390 V coating contained less DCPD and thus had a higher HA/DCPD ratio and a Ca/P ratio close to the ideal value of HA. The flower-like structures consisted mainly of HA-containing compounds, while the plate-like structures consisted of DCPD. The 450 V coatings consisted mainly of Ti, TiO_2_-A, TiO_2_-R, and CaTiO_3_.
3. The contact angle (CA) of the ground Ti surface was around 47°. By contrast, the CA values of the 330 V and 390 V coatings, with flower-like/plate-like and flower-like structures on the surface, respectively, were close to 0°, while the CA value of the 450 V sample was approximately 3°. In other words, the Ti substrate and MAO coatings had sufficient hydrophilicity, with the flower-like/plate-like and flower-like structure surfaces having a better wettability than the traditional MAO porous surface.
4. The 330 V, 390 V, and 450 V coatings were completely covered with apatite after 7 days, 3 days, and 7 days immersion in SBF, respectively. However, no apatite was formed on the surface of the bare Ti substrate. The Ca/P ratios of the 330 V and 390 V samples increased from 1.23 to 1.74 and 1.51 to 1.66, respectively, following immersion in SBF for 7 days. By contrast, the Ca/P ratio of the 450 V sample fell from 4.13 to 1.91. The bioactivity of the MAO coatings was significantly correlated with the HA and CaTiO_3_ contents of the coating prior to immersion in SBF.
5. In MG-63 cell culture tests, the flower-like/plate-like structure coatings (330 V) and flower-like structure coatings (390 V) showed partial cell coverage and protruded filopodia after 3 h. The 450 V coating and bare Ti substrate exhibited obvious cell expansion after 24 h, but no filopodia growth. The samples are ranked in order of decreasing in vitro biocompatibility as follows: 390 V > 330 V > 450 V > Ti substrate.

## Figures and Tables

**Table 1:** Inorganic ion concentrations of SBF and human blood plasma.

(mM)	Na^+^	K^+^	Ca^+2^	Mg^+2^	Cl^−^	HCO_3_^−^	HPO_4_^−2^
SBF	142.0	5.0	2.5	1.5	148.8	4.2	1.0
Blood	142.0	5.0	2.5	1.5	103.0	27.0	1.0

## Data Availability

Not applicable.
